# Twenty Years of Investigating Angiotensin Activity: An Overview

**Published:** 1991-03

**Authors:** Edward C. Osborn, J. Campbell Mackenzie, Linda M. Fearn

**Affiliations:** Department of Renal Medicine, Southmead Hospital, Westbury-on-Trym, Bristol BS10 5NB; Department of Renal Medicine, Southmead Hospital, Westbury-on-Trym, Bristol BS10 5NB; Department of Renal Medicine, Southmead Hospital, Westbury-on-Trym, Bristol BS10 5NB

## Abstract

A short account of the renin-angiotensin-aldosterone system is given with emphasis on the relationship between the octapeptide angiotensin II and its decapeptide precursor angiotensin I. Although the decapeptide is generally assumed to have no biological activity, extensive investigations have indicated that such an assumption is almost certainly unjustified. The implications of the findings are briefly outlined and discussed.


					West of England Medical Journal Volume 106 (i) March 1991
Twenty Years of Investigating Angiotensin Activity:
an Overview
Edward C. Osborn PhD, J. Campbell Mackenzie
JFRCP (Ed), Linda M. Fearn BSc,
^Department of Renal Medicine, Southmead Hospital,
Westbury-on-Trym, Bristoj^BSlO 5NB.
SUMMARY
r~A short account of the renin-angiotensin-aldosterone system is
given with emphasis on the relationship between the octapeptide
angiotensin II and its decapeptide precursor angiotensin I.
Although the decapeptide is generally assumed to have no
biological activity, extensive investigations have indicated that
such an assumption is almost certainly unjustified. The
implications of the findings are briefly outlined and discussed. .
INTRODUCTION
Since the observation of Tigerstedt and Bergman, reported in
1898, that the kidney contains a highly pressor substance that they
named renin1 there has been intense scientific and clinical interest
concerning its in vivo operation. Renin itself does not raise the
blood pressure directly, being an enzyme that acts on its substrate
to release the decapeptide angiotensin 1 by the splitting of a
leucyl-leucine bond.2-3 The generally assumed active component
of the renin-angiotensin (RA) system, the octapeptide angiotensin
II, then results from the loss of histidyl-leucine from the carboxy
end of angiotensin I by the action of an endopeptidase,
angiotensin converting enzyme2,3 now generally referred to as
ACE. Thus:
Renin ACE
Renin substrate  ? Angiotensin I ?? Angiotensin II
(a, - globulin) (decapeptide) (octapeptide)
With the involvement of aldosterone also (as discussed by
Gross, Brunner and Ziegler4) the overall system is known as
RAA.
THE EMPHASIS ON ANGIOTENSIN II
The almost universal belief that angiotensin II is the be-all and
end-all of both RA and RAA activity is based on the findings of
Skeggs , Kahn and Shumway in the mid '50s (as indicated in 5;
reference 33) that, whereas angiotensin II immediately causes
profound increases in renal vascular resistance in the isolated
perfused dog kidney, by strong contrast angiotensin I has a
negligible straightway effect on it. It was therefore concluded that
conversion of the deca- to the octa- peptide by a circulating
plasma enzyme is necessary for pressor activity within the kidney
and presumably at other vascular receptor sites for angiotensin II.
The supposed plasma conversion then held sway for some fifteen
years until several groups, employing widely differing
experimental approaches, showed that ACE activity in the
circulating blood could not account for the rapid and efficient in
vivo generation of angiotensin II. It became evident that the lungs
were important for the formation of the octapeptide and its
immediate release into the pulmonary vein but that ACE activity
was presumably not confined to that organ (as indicated in 5; 4).
The intra-pulmonary conversion satisfied almost all investigators
in both the RA and RAA fields since it did not disturb their
cherished conviction that angiotensin II was omnipotent, a belief
no doubt buttressed by its more ready availability than that of
angiotensin I. However the shift of emphasis from plasma to
tissue convertibility was profoundly to affect ideas concerning the
ability of drugs to modify RA (and RAA) operation by the
inhibition of ACE activity at tissue level, resulting in blood
pressure reduction.
THE EMERGENCE OF ANGIOTENSIN I
Until 1970 synthetic angiotensin I was difficult to obtain although
by then a few studies with its use had been reported. The
subsequent greater availability of it tended to confirm the general
conviction that angiotensin II is the active component of the RA
system. However strong doubts were expressed in some quarters;
these especially concerned the nature of pressor responses
established in anaesthetised sheep6 and those in conscious rabbits
observed by Munday, Noble and Rowe at Southampton
University (refer 5; 28-31) and also the suggestion that
angiotensin I itself could have an antidiuretic effect on the
peritubular capillaries that is plasma [NaCl] dependent.7
ANGIOTENSIN RECEPTOR DISTRIBUTION
The importance of the pulmonary circulation in the conversion of
angiotensin I to angiotensin II has been established in extensive
studies.6,8 These and many other investigations (refer 5; 1-3, 5, 7)
have highlighted the strikingly contrasting manner whereby the
two peptides are handled by the lungs. Thus an Acceptance/
Rejection (AR) situation occurs in that organ in which there is a
highly effective removal of the decapeptide from the bloodstream
but with free traverse through the pulmonary circulation for the
octapeptide itself. However the possibility that differential
treatment of the angiotensins happens elsewhere in the body has
not received a great deal of attention. That the peptides have
complex relationships within the kidney has been argued
recently5- 9 a matter that concerns the question whether common
receptors invariably occur in the renal vasculature - ie an
Acceptance/Acceptance (AA) situation throughout - or is there
involvement of AR and/or RA receptor sites?
The pulmonary generation of angiotensin II clearly ensures a
widespread distribution of the octapeptide throughout the
systemic circulation. It is therefore not surprising that intravenous
injections of angiotensins I and II produced almost identical
effects concerning increases in blood pressure6 reductions in renal
blood flow - RBF10 and water shifts into renal venous blood.1112
However, with injection into the left ventricle (LV) - although the
pressor responses to the angiotensins are not too dissimilar - the
immediate ability of the decapeptide to lower RBF10 and to
increase the relative plasma content of blood draining from the
kidney11,12 is very much less than that of the octapeptide.
The abilities of the intravenously injected peptides to produce
such similar effects could well be considered to support the belief
that all biological effects attributable to renin-angiotensin
operation reside in angiotensin II. However it is not possible to
reconcile such a viewpoint with the observed renal venous plasma
[creatinine] changes that occur with injection of the angiotensins
into the general circulation. Thus, whereas with intravenous
injections of angiotensin II (and also with injection into the LV, ie
with direct presentation to the kidney) the [creatinine] of renal
venous plasma is greatly raised, intravenously injected
angiotensin 1 invariably considerably lowers the plasma creatinine
levels.5,9 There is therefore the paradoxical situation that, although
with intravenous administration both peptides similarly affect
blood pressure, RBF and the relative plasma content of blood
from the renal vein, their effects on the creatinine content of blood
leaving the kidney are diametrically opposed. The explanation
probably involves a complex interplay between angiotensins I and
II in the kidney that importantly concerns the question of the
distribution of intra-renal angiotensin receptors and also that of
blood pressure and blood and water flows.'1 These ideas are highly
pertinent to the question of acute renal failure precipitated in
patients by ACE inhibition; this is a not uncommon effect of such
treatment.13'14
Because arterial plasma renin and angiotensin I levels are
invariably much increased by ACE inhibition - with a
concomitant lowering of angiotensin II concentrations (refer 5;
West of England Medical Journal Volume 106 (i) March 1991
80-82) the questions of adrenal receptors for the peptides is
currently of great interest. With infusions over several hours into
the adrenal arteries of conscious sheep15 the decapeptide was
found, on average, to be very much less effective than was the
octapeptide in stimulating the zona glomerulosa to secrete
aldosterone. When considered together with a later report that, in
vitro, bovine adrenal gland receptors showed "low or negligible
binding of angiotensin II precursors such as angiotensin I and the
tetradecapeptide sequence of renin substrate..."16 there is clearly a
strong possibility that a RA situation exists in the adrenals.
The probable existence of an AA situation in the hind-limbs has
been indicated by extensive studies utilizing angiotensins 1 and II
and a variety of experimental techniques. This has enabled one
aspect of the relationship between the peptides to be closely
examined, especially concerning the effects of ACE inhibition (as
discussed in 5; 39, 86). However, despite the ability of the
angiotensins to lower hind-limb blood flow with direct
presentation to the limbs - the decapeptide is mostly reckoned to
be 10 to 40% as potent as the octapeptide - large increases in
flow can readily be achieved with injection of either peptide into
the general circulation (refer 5; 23) indicating the negligible
significance of local vasoconstriction.
ANGIOTENSIN - PRODUCED ARTERIAL PLASMA
[K+] RISES AND [Na+], [CI ] FALLS
The ability of angiotensin II simultaneously to raise the
circulating plasma [K+| and to lower the levels of the sodium and
chloride ions was initially reported by Healy, Elliott and
Harrison17 who employed one hourly intravenous infusions. A
similar pattern of arterial plasma [electrolyte) response was also
evident with 6 minute intravenous administration of the
octapeptide18- 19 and also with that of the decapeptide;9- ls the
pressor catecholamine noradrenaline, however, only minimally
released potassium ions into the circulation and did not affect the
plasma levels of the sodium and chloride ions.18 These various
observations importantly relate to the well documented influence
of Na+, K+ concentration ratios in influencing aldosterone
secretion1-'1 and the relative inability of noradrenaline - in
comparison with the effects of angiotensin II - to enhance the
release of the steroid from the adrenal cortex.15-20 The findings
with use of the angiotensins were surprising since, until the report
of Healy and his colleagues, it was almost universally considered
that they had little, if any, ability to influence arterial plasma
electrolyte concentrations. This is particularly so because the
liberated potassium was readily detectable at infusion rates of
only 0.5 to 2|ig per minute17-18-19
Further reports concerned the use of the released K+ ions as a
possible marker for the intra-cellular transfer of Ca2+ ions21 and
the question of lung involvement respecting the observed pattern
of plasma [K+] increases22 (when it was concluded that the most
likely source of the potassium was the resistance arterioles of the
systemic circulation). Another interesting aspect involves the pos-
sibility that the additional circulating potassium ions could have a
vasodilator effect.23 The importance of establishing the cellular
electrolyte distribution changes associated with the use of highly
pressor compounds requires no emphasis because of the involve-
ment of such variations in the genesis and maintenance of
hypertension.24
CONCLUSIONS
The findings of Departmental investigations during the past 20
years - that concerned the effects of angiotensins I and II on (a)
blood pressure (b) RBF and intra-renal water, electrolyte, creatinine
and urea distribution and creatinine elimination from arterial plasma
(c) hind-limb blood flow and (d) arterial plasma electrolyte levels ?
have extensive physiological and clinical implications. They do not
support the almost universal conviction that the effects of RA, and
RAA, operation are mediated solely through angiotensin il but
strongly indicate a vital role for its precursor angiotensin I in
affecting blood pressure in a volume-related manner that is [NaCl ]
dependent and mediated via the peritubular capillaries.
ACKNOWLEDGEMENTS
Generous financial assistance was provided by the Bristol Kidney
Fund, the Sir Halley Stewart Trust, Westbury Scientific Enterprise
Limited and the Dawn James Charitable Foundation.
REFERENCES
1. TIGERSTEDT, R., BERGMAN, P.G. (1898) Niere unci Kreislauf.
Skand. Arch. Physiol. 8, 223-246.
2. PEART, W.S. (1965) The renin-angiotensin system. Pharmacol.
Rev. 17, 143-182.
3. REID. I.A., MORRIS, B.J., GANONG, W.E. (1978) The renin-
angiotensin system. Ann. Rev. Physiol. 40, 377-410.
4. GROSS, F., BRUNNER, H.,' ZIEGLER, M. (1965) Renin-
angiotensin system, aldosterone and sodium balance. Recent Progr.
Harm. Res. 21,119-177.
5. OSBORN, E.C., FEARN, L.M., FRANCIS, R.J., MACKENZIE,
J.C., WILSON, J. (1991) Receptors for angiotensins I and II: their
relevance to renal haemodynamics, blood pressure control and hind-
limb flow. Med. Hypoth. In press, (a longer complementary version
available on request).
6. OSBORN, E.C., TILDESLEY, G? PICKENS, P.T. (1972) Pressor
response to angiotensin I and angiotensin II: the site of conversion of
angiotensin I. Clin. Sci. 43, 839-849.
7. OSBORN, E.C., FEARN, L.M., MACKENZIE, J.C.. MASON. O.F.,
RIGBY, G..V., RODGERS, M.W., WILSON, J. (1987) Do
angiotensin-induced changes in sheep renal venous blood and
plasma composition indicate a reversal of glomerular filtration? Med.
Hypoth. 23, 137-148.
8. OSBORN, E.C., MACKENZIE, J.C., RIGBY, G.V., WILTON. A.,
SUGDEN, P.L. (1977) Radioimmunoassay of unprocessed sheep
blood extracts to follow angiotensin metabolism. Am. ./. Physiol.
233, H527-H534.
9. OSBORN, E.C., FEARN, L.M., FRANCIS. R.J., MACKENZIE,
J.C., MASON, O.F., RIGBY, G.V., WILSON, J. (1989) Arterial
plasma creatinine and K+ levels with use of exogenous creatinine
and intravenously infused angiotensin I in conscious sheep. Med.
Sci. Res. 17, 677-679.
10. OSBORN, E.C., TILDESLEY, G? LEACH, K.G., RIGBY, G.V.
(1974) Effects of angiotensin I and angiotensin II on renal blood
flow in sheep. Am../. Physiol. 226,518-522.
11. OSBORN, E.C., MACKENZIE, J.C.. RIGBY, G.V. (1976) The
effects of angiotensin I on the relative cellular content of renal vein
blood in sheep. IRCS Med. Sci. 4, 358.
12. OSBORN, E.C., MACKENZIE, J.C.. RIGBY, G.V. (1975) The
effects of angiotensin II, noradrenaline and autonomic blockade on
the relative cell content of renal vein blood in sheep. IRCS Med. Sci.
3, 289.
13. HOLLENBERG. N.K. (1987) The treatment of renovascular
hypertension: surgery, angioplasty, and medical therapy with
converting enzyme inhibitors. Am../. Kidney Dis. 10, 52-60.
14. FERNER, R.E. (1990) Adverse effects of angiotensin - converting
enzyme inhibitors. Adverse Drug Reaction Bull. No. 141, 528-531.
15. BLAIR-WEST, J.R., COGHLAN, J.P., DENTON, D.A., CODING.
J.R., MUNRO, J.A.. PETERSON. R.E., WINTOUR. M. (1962)
Humoral stimulation of adrenal cortical secretion../. Clin. Invest. 41,
1606-1627.
16. CATT, K.J., AGUILERA, G., CAPPONI, A., FUJITA, K.,
SCHIRAR, A., FAKUNDING, J. Angiotensin II receptors and
aldosterone secretion../. Endocrinol. 81, 37P-48P, 1979.
17. HEALY, J.K., ELLIOTT, A.J., HARRISON, L.C. (1974) Effects of
angiotensin on plasma electrolyte concentrations in rabbits and in
man. Clin. Sci. Mot. Med. 46, 19-36.
18. OSBORN, E.C., SUGDEN, P.L., MACKENZIE, J.C., AITKEN,
D.M., CHAPMAN. I.D.. HOWES, S? MASON, O.F., RIC.BY, G.V.,
WILSON, J. (1985) Influence of angiotensin II on the concentration
of arterial plasma electrolytes in anaesthetized sheep../. Endocrinol-
104,143-148.
19. DYSON, C.E., FEARN, L.M., FRANCIS, R.J., JOHNSON, J-L-
KENDALL, H.J., MACKENZIE, J.C., OSBORN, E.C.,
UNDERHILL, D.G. (1988) Arterial plasma electrolyte
concentrations with short-term intravenous infusions of angiotensin
II in conscious sheep. Med. Sci. Res. 16, 817-818.
20. FEARN, L.M., JAMES, H.C.F., MACKENZIE, J.C., OSBORN,
E.C. (1987) The relationship between angiotensin and aldosterone
activity: the implications of angiotensin II - induced potassium
release. Med. Hypoth. 24, 329-330.
Twenty Years of Investigating Angio-
tensin activity continued from page 6
21. FEARN. L.M., MACKENZIE, J.C., OSBORN, E.C. (1987) Arterial
plasma [K+] increases produced by angiotensin II: a marker for
intracellular shifts of Ca2+ ions? Med. Hypoth. 24, 241-242.
22. FEARN, L.M., MACKENZIE, J.C., OSBORN, E.C. (1988) The
question of lung involvement in angiotensin II - induced rises in
circulating plasma [K+J and arterial blood pH. Med. Hypoth. 25,
61-63.
23. FEARN, L.M., OSBORN, E.C. (1987) Arteriolar resistance and
plasma [K+] increases produced by angiotensin II: the implications
of potassium - induced depressor responses. Med. Hypoth. 23,
441-442.
24. SEMPLE, P.F., LEVER, A.F. (1986) Editorial - Glimpses of the
mechanisms of hypertension. Br. Med. J. 293, 901-901.

				

